# Engineering the *N*-glycosylation pathway of *Nicotiana tabacum* for molecular pharming using CRISPR/Cas9

**DOI:** 10.3389/fpls.2022.1003065

**Published:** 2022-09-08

**Authors:** Kathrin Göritzer, Melanie Grandits, Clemens Grünwald-Gruber, Rudolf Figl, Sébastien Mercx, Catherine Navarre, Julian K-C. Ma, Audrey Y-H. Teh

**Affiliations:** ^1^Hotung Molecular Immunology Unit, Institute for Infection and Immunity, St. George’s University of London, London, United Kingdom; ^2^Core Facility Mass Spectrometry, University of Natural Resources and Life Sciences, Vienna, Austria; ^3^Louvain Institute of Biomolecular Science and Technology, UCLouvain, Louvain-la-Neuve, Belgium

**Keywords:** *N*-glycosylation, glycoengineering, molecular pharming, recombinant protein production, CRISPR/Cas9, genome editing, *Nicotiana tabacum* using CRISPR/Cas9, NtFX-KO

## Abstract

Molecular pharming in plants offers exciting possibilities to address global access to modern biologics. However, differences in the *N*-glycosylation pathway including the presence of β(1,2)-xylose and core α(1,3)-fucose can affect activity, potency and immunogenicity of plant-derived proteins. Successful glycoengineering approaches toward human-like structures with no changes in plant phenotype, growth, or recombinant protein expression levels have been reported for *Arabidopsis thaliana* and *Nicotiana benthamiana*. Such engineering of *N*-glycosylation would also be desirable for *Nicotiana tabacum*, which remains the crop of choice for recombinant protein pharmaceuticals required at massive scale and for manufacturing technology transfer to less developed countries. Here, we generated *N. tabacum* cv. SR-1 β(1,2)-xylosyltransferase (*XylT*) and α(1,3)-fucosyltransferase (*FucT*) knockout lines using CRISPR/Cas9 multiplex genome editing, targeting three conserved regions of the four *FucT* and two *XylT* genes. These two enzymes are responsible for generating non-human *N*-glycan structures. We confirmed full functional knockout of transformants by immunoblotting of total soluble protein by antibodies recognizing β(1,2)-xylose and core α(1,3)-fucose, mass spectrometry analysis of recombinantly produced VRC01, a broadly neutralizing anti-HIV-1 hIgG1 antibody, and Sanger sequencing of targeted regions of the putative transformants. These data represent an important step toward establishing *Nicotiana tabacum* as a biologics platform for Global Health.

## Introduction

Plants are emerging as an alternative manufacturing platform to mammalian cells for manufacturing high-value recombinant biopharmaceuticals such as monoclonal antibodies. The plant platform can be cost-effective, easily scalable, and are able to carry out complex post-translational modifications such as complex *N*-glycosylation (Stoger et al., 2014; Ma et al., 2015; Murad et al., 2020). Plants produce similar glycan structures to those found on mammalian glycoproteins, but the glycosylation repertoire in the Golgi of plants is much reduced. They lack pathways for galactosylation, sialylation, core α(1,6)-fucosylation, bisecting GlcNAc and branching of *N*-linked glycans which are present in mammalian cells. Perhaps more significantly, plant-specific modifications such as β(1,2)-xylose and core α(1,3)-fucose are present on plant glycoproteins. Both of these *N*-glycan modifications have been linked with an increased risk for immunogenicity and adverse allergic reactions in humans (Bardor et al., 2003; Jin et al., 2008; Paulus et al., 2011), although the significance of these risks is not established (McCormick et al., 2003; Shaaltiel and Tekoah, 2016). Nevertheless, regulatory caution makes their elimination desirable. Furthermore, the removal of these residues improves effector functions such as antibody-dependent cellular cytotoxicity (ADCC) in antibodies (Stelter et al., 2020). Consequently, there has been a lot of effort to eliminate and modify the *N*-glycosylation pathway in a variety of plant species such as *Arabidopsis thaliana*, *Nicotiana benthamiana,* and *Nicotiana tabacum* BY-2 suspension cells using different strategies such as RNAi silencing (Strasser et al., 2008), and more recently targeted CRISPR/Cas9 nucleases (Jansing et al., 2019).

Plants such as *N. benthamiana* tolerate the removal of plant-specific complex *N*-glycans very well, as no obvious phenotypes have been described in plants with their xylosyltransferase and fucosyltransferase silenced or knocked out (Strasser et al., 2004, 2008; Jansing et al., 2019). Recombinant proteins from these plants also display very homogeneous glycosylation profiles with primarily GnGn *N*-glycans, which is the preferred glycoform for engineering of glycan extensions and introduction of mammalian-type complex *N*-glycan modifications (Castilho et al., 2010; Strasser et al., 2014). Although many successful glycoengineering approaches toward human-like structures have been reported for *Arabidopsis thaliana* and *Nicotiana benthamiana*, advances in *Nicotiana tabacum* have lagged behind (Ma et al., 2003). With a larger biomass, established techniques for stable gene transformation, and simple cultivation conditions, *N. tabacum* is the crop of choice for manufacturing recombinant protein at massive scale and technology transfer to resource poor settings (Ma et al., 2003). Humanization of the *N*-glycosylation pathway may be an additional important step to further advance *Nicotiana tabacum* for molecular farming of high-value, non-smoking products.

*Nicotiana tabacum* is an allotetraploid resulting from a cross between *N. sylvestris* and *N. tomentosiformis*. The genome of the cultivar Petit Havana SR-1 (henceforth known as SR-1) commonly used for recombinant protein production (Teh et al., 2014; Ma et al., 2015) has not been fully annotated yet. It is expected that it contains two β(1,2)-xylosyltransferase (*XylT*) as well as four α(1,3)-fucosyltransferase (*FucT*) isoforms corresponding to the parental genes, which are responsible for addition of plant-specific xylose and core-fucose *N*-glycan modifications. We initially designed several single guide RNAs (sgRNAs) for CRISPR/Cas9-mediated knockout of *N. tabacum* cv. SR-1 *XylT* and *FucT*, targeting orthologous regions of *N. benthamiana XylT* and *FucT* which has previously been successfully knocked out (Jansing et al., 2019). Although these constructs achieved high levels of editing efficiencies in the targeted regions, they did not yield a functional *XylT* and *FucT* knockout *N. tabacum* cv. SR-1 line. Later, Mercx et al. (2017) have described the successful multiplex CRISPR/Cas9-mediated knockout of four *FucT* genes and two *XylT* genes in *N. tabacum* BY-2 suspension cells by targeting 12 alleles with sgRNAs specific to three conserved regions of the *XylT* exon 1 and six in *FucT* exon 3. We successfully employed the same CRISPR/Cas9 construct to generate *N. tabacum* cv. SR-1 plant lines for the stable production of recombinant proteins completely devoid of core α(1,3)-fucose and β(1,2)-xylose residues.

## Materials and methods

### Cas9 and sgRNA plasmid construction

For the pFGC-FucT and pFGC-XylT constructs targeting *N. tabacum* cv. SR-1 *FucT and XylT* respectively, putative *N. tabacum* cv. Petit Havana SR-1 *FucT* or *XylT* mRNA sequences were obtained through NCBI BLAST (Altschul et al., 1990) using the *N. benthamiana* orthologs as a template. Five *N. tabacum FucT* predicted mRNA variants were found, of which three shared high similarity and the other two (GenInfo Identifier 1025362229 and 1025193416) were grouped together (Supplementary Figure S1). Furthermore, five *N. tabacum XylT* predicted mRNA variants were found and all *XylT* mRNA showed high similarity (Supplementary Figure S2). The location of introns in the regions of interest were identified by gene walking from known mRNA sequences.

Protospacer elements were designed manually using the criteria GN_20_-GG (for U6 promoters) and checked using the online tool CRISPR-P (Lei et al., 2014). Twelve protospacer elements were selected for *FucT* (Supplementary Figure S1) and six for *XylT* (Supplementary Figure S2). The individual protospacer elements were synthesized by GeneArt, United States, and assembled into separate sgRNA expression cassettes as per Li et al. (2013) using pUC119-gRNA (Addgene plasmid #52255) as template. The sgRNA expression cassettes were then cloned into the plant transformation vector pFGC-pcoCas9 (Addgene plasmid #52256) using flanking AscI and PacI restriction sites. The vectors were then transformed into *Agrobacterium tumefaciens* strain GV3101. To test the cutting efficiency of the individual constructs, *A. tumefaciens* harboring the Cas9 and sgRNA cassettes were transformed into 8-week-old *N. tabacum* cv. SR-1 plants. DNA was isolated from infiltrated leaves 3 days-post-infection (dpi) and the regions targeted by the sgRNAs were amplified using primers indicated in Supplementary Figures S1, S2 using the Extract-N-Amp™ Plant Tissue PCR kit (Merck, Germany). The PCR products were then used for Indel Detection by Amplicon Analysis (IDAA) as previously described (Yang et al., 2015). sgRNA that showed high cutting efficacy (F2, F5-7, F11 for *FucT* and X2, X4-6 for *XylT*) were used to generate multiplex constructs pFGC-FucT and pFGC-XylT constructs, respectively, (Li et al., 2013, 2016).

The second CRISPR/Cas9 construct used, targeting *N. tabacum* BY-2 *FucT* and *XylT* genes (henceforth called pFGC-LFX), has been described in detail previously by Mercx et al. (2017). In short, the sgRNAs were selected manually based on the annotated *XylT* and *FucT* genes from *N. tomentosiformis* and *N. sylvestris XylTA* (NM_001324669), *XylTB* (NM_001325611), *FucTA* (XM_016657530), *FucTB* (XM_016620229), *FucTC* (NM_001324945) and *FucTD* (XM_016585847). The polycistronic tRNA-gRNA was synthesized by GenScript and cloned into the SbfI restriction sites of pFGC-pcoCas9 binary vector (Li et al., 2013). The vector was transformed into *A. tumefaciens* LBA4404virG (van der Fits et al., 2000) by electroporation.

### Stable transformation and plant cultivation

*Agrobacterium tumefaciens* containing sgRNAs constructs were grown overnight in Yeast Extract Mannitol (YEM) medium (0.04% Yeast extract, 10 g/L mannitol, 1.7 mM NaCl, 0.8 mM MgSO_4_, 2.2 mM K_2_HPO_4_, pH7.0) containing 50 μg/ml Kanamycin and 25 μg/ml Rifampicin. Growth medium was removed by centrifugation at 1,600 rpm for 10 min and *A. tumefaciens* were resuspended in liquid Murashige and Skoog (MS; Murashige and Skoog, 1962) medium. Leaf disks (1 cm^2^) were prepared from 4-week-old *N. tabacum* cv. SR-1 grown under sterile conditions on MS agar and immersed in bacterial suspension for 20 min. The leaf disks were blot dried on filter paper followed by incubation on shoot-inducing medium (SIM) consisting of MS medium, 0.1 μg/ml alpha-Naphthaleneacetic acid (NAA, Sigma) and 1 μg/ml Benzylaminopurine (BAP, Sigma) in the dark at 23°C for 2 days. The leaf disks were then transferred to SIM containing 3 μg/ml Glufosinate-ammonium (Pestanal^®^, Sigma) to select for transformed cells and 300 μg/ml Timentin (Ticarcillin/Potassium Clavulante mixture 15:1; Melford Laboratories, United Kingdom) to eliminate *Agrobacteria*. Regenerated shoots appeared 5–8 weeks after transformation and were rooted on MS medium containing 300 μg/ml Timentin. After another 4–6 weeks, rooted plantlets were transferred to soil and maintained in the greenhouse with a 16/8-h day/night cycle at 24°C–28°C.

### Transient expression and purification of VRC01 IgG from detached *Nicotiana tabacum* leaves

Leaves from mature *N. tabacum* cv. SR-1 transformants from the T_1_ generation were used for transient expression of the broadly neutralizing anti-HIV-1 antibody VRC01 (Wu et al., 2010; Teh et al., 2014). VRC01 light (kappa) and heavy (gamma) chain genes in MIDAS-P modular plant expression vector (Pinneh et al., 2022) was used for the transient expression experiments. Recombinant *A. tumefaciens* harboring the construct were grown to an OD_600_ of 2–4. The culture was diluted to a final OD_600_ of 0.2 in infiltration solution (IS; 10 mM MgSO_4_, 10 mM MES, pH5.6) and incubated at room temperature for a minimum of 30 min with 200 μM acetosyringone. Detached leaves were infiltrated by vacuum infiltration and incubated in a humidified atmosphere in the green house with a 16/8-h day/night cycle at 24°C–28°C. After 4 days, infiltrated leaves were harvested, snap-frozen in liquid nitrogen, and ground with a pestle and mortar. 300 μl per 100 mg leaf fresh weight (LFW) of extraction buffer (1 × PBS with 0.1% Tween20) was mixed to homogenized leaf material to extract the total soluble protein. The crude leaf extract was centrifuged at 25,000×*g* for 20 min at 4°C, passed through a Miracloth filter (Merck Millipore, Germany), and loaded on a chromatography column packed with Pierce™ Protein A resin (Thermo Fisher Scientific, United States) pre-equilibrated with 5 column volumes (CVs) of binding buffer (1 × PBS, pH 7.4). After washing with 5 CVs binding buffer, bound proteins were eluted with 100 mM glycine pH 2.7. The eluted fractions were neutralized with 1 M Tris–HCl, pH 9.0 and subsequently dialyzed against 1 × PBS using Slide-A-Lyzer Cassettes (Molecular cut-off 3.5 kDa; Thermo Scientific, United States).

### Immunoblot analysis

Plant leaf tissue was homogenized in 2 μl 1 × PBS per mg LFW using 3 mm chrome steel ball bearings and a Mixer Mill MM400 (Retsch, Castleford, United Kingdom). Crude extract was centrifuged at 20,000×*g* for 15 min and clarified extracts were resolved on a NuPage 4%–12% Bis-Tris gel (Life Technologies, Paisley, United Kingdom) and transferred onto a nitrocellulose membrane. The membrane was blocked with blocking buffer containing 5% skimmed milk in 1 × TBS + 0.05% Tween.

To analyze the fucosyl- and xylosyl *N*-glycan composition in total soluble protein, blots were incubated with either 1:5,000 rabbit anti-xylose or anti-fucose antisera (both from Agrisera, Sweden), followed by 1:10,000 peroxidase-conjugated polyclonal anti-rabbit antisera (Sigma, United States). Detection was performed using Amersham ECL substrate (GE Healthcare, United Kingdom) visualized by a G:BOX F3 (Syngene, United Kingdom).

To analyze the fucosyl- and xylosyl *N*-glycan composition of purified recombinant IgG, 200 ng protein were loaded onto a NuPage 4%–12% Bis-Tris gel. SDS-PAGE and Western blotting was performed as described above. Membranes were subsequently probed 1:5,000 with HRP-labeled anti-IgG H + L chain antibody (31410, Thermo Fisher Scientific, United States) or anti-xylose and anti-fucose antibodies and developed as above.

### Mass spectrometry analysis of IgG glycosylation

Twenty microgram of purified protein was reduced by adding dithiothreitol (7.5 mM final concentration) in 100 mM ammonium bicarbonate buffer pH ~8, and incubated at 56°C for 45 min. *S*-alkylation was performed using Iodoacetamide (7.5 mM final concentration). Proteins were precipitated by adding 40 μl per 10 μg protein of ice cold acetone, the pellets washed with 80% acetone, and dried in a SpeedVac vacuum concentrator. The pellets were re-dissolved in 50 mM ammonium bicarbonate and Trypsin (Promega, United States) to 1 g/L and a protein to Trypsin ratio of 60:1. The sample was incubated overnight at 37°C. If required, samples were additionally digested with the endoprotease GluC (Promega, United States). Glycopeptides were then analyzed by capillary reversed-phase chromatography and electro-spray MS using a Agilent Series 6560 LC-INS-QTOF instrument as described previously (Göritzer et al., 2017).

### Analysis of genome modifications

Genomic DNA was extracted from leaves of stable transgenic transformants using Extract-N-Amp™ Tissue PCR kit (Merck, Germany) according to the manufacturer’s protocol. PCR was performed using primers (Supplementary Table S2) flanking the targeted regions. The PCR products were purified using QIAquick PCR purification kit (QIAGEN, Germany) and the amplicons were sequenced by Sanger sequencing (Genewiz, United Kingdom). The results were analyzed using the Synthego ICE analysis tool. If the sequencing results were ambiguous, the PCR products were cloned in pTOPO from the Zero Blunt^®^ TOPO^®^ PCR Cloning Kit (Thermo Fisher Scientific, United States) and 5–10 clones were sequenced individually for each sample.

## Results

### Generation, screening and selection of FucT and XylT knockout plant lines

Several pFGC-CRISPR/Cas9 constructs putatively targeting α(1,3)-fucosyltransferase (*FucT*) and β(1,2)-xylosyltransferase (*XylT*) of the non-sequenced *N. tabacum* cv. SR-1 were generated in house based on mRNA sequences obtained from BLAST alignment using *N. benthamiana* orthologs as well as gene walking. The cutting efficiency of twelve individual sgRNAs targeting *FucT* (Supplementary Figure S1) and six targeting *XylT* (Supplementary Figure S2) were tested. The sgRNA targeted regions were amplified using primers indicated in Supplementary Figures S1, S2 for subsequent Indel Detection by Amplicon Analysis (IDAA). sgRNAs that showed high cutting efficiency (F2, F5-7, F11 for *FucT*; X2, X4-6 for *XylT*; Supplementary Table S1) were used to generate multiplex constructs pFGC-FucT and pFGC-XylT (Li et al., 2013, 2016). After *Agrobacterium*-mediated transformation of leaf explants, putative knockout lines were screened by amplification of targeted region with primers indicated in Supplementary Figures S1, S2, followed by Sanger sequencing of targeted regions and analysis of introduced mutations using the Synthego ICE analysis tool (Conant et al., 2022). The editing efficiencies of the T_2_ generation of *FucT* and T_3_ generation of *XylT* putative knockout lines reached 90% and 30%, respectively, (Supplementary Figures S3A,B). Although high knockout scores, especially for *FucT* transformants, were observed after several generations, immunoblotting using anti-α(1,3)-fucose and anti-β(1,2)-xylose antibodies displayed residual plant-specific *N*-glycan modifications on endogenous plant proteins of both lines (Supplementary Figures S4A,B).

### Generation, screening and selection of NtFX-KO plants

Mercx et al. (2017) showed the successful CRISPR/Cas9 genome editing of *N. tabacum* BY-2 suspension cells diploid of *XylT* and *FucT,* using the construct pFGC-LFX that employed the same polycistronic cassette strategy and vector backbone. However, sgRNAs were designed based on the annotated *XylT* and *FucT* genes from *Nicotiana tomentosiformis* and *Nicotiana sylvestris.* The construct contains three sgRNAs targeting three conserved regions of the *XylT* exon 1 and six sgRNAs targeting three conserved regions of *FucT* exon 3 to increase the likelihood of mutating all isoforms (Li et al., 2013; Xie et al., 2015). Since the genome of the *N. tabacum* cv. SR-1 has not been annotated, sequence homology of the targeted regions in the *XylT* and *FucT* genes with the *N. tabacum* BY-2 cell line was confirmed by amplification of these regions using primers that were designed based on the draft genome of *N. tabacum* cultivar K326 and the BY-2 plant cell lines (Supplementary Table S2).

**Figure 1:**
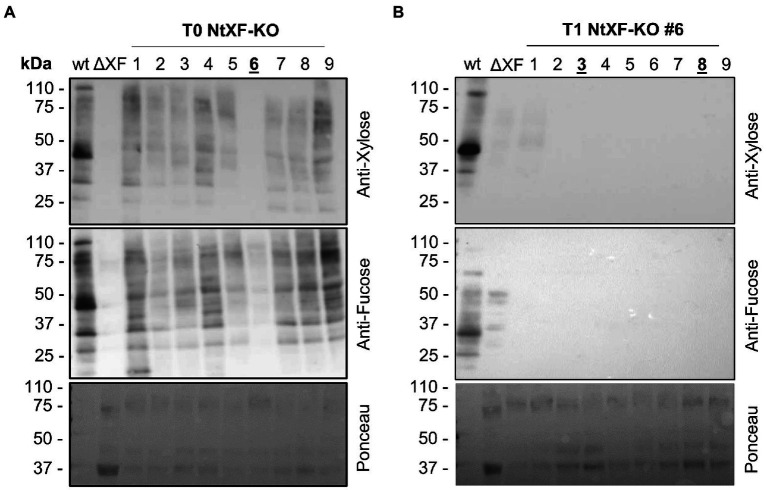
Western blot analysis of total soluble protein from the T_0_ **(A)** and T_1_ **(B)** generations of transgenic NtFX-KO lines. About 10 μg of total soluble protein from wild-type *Nicotiana tabacum* cv. SR-1 (wt), putative NtFX-KO lines and *N. benthamiana* ΔXF/FT plants (ΔXF; kindly provided by BOKU Vienna), were loaded and incubated with either rabbit anti-α(1,3)-fucose or anti-β(1,2)-xylose antisera, followed by HRP-labeled goat-anti-rabbit H + L antisera. Ponceau staining of probed nitrocellulose membranes was used as loading control.

Fifteen transgenic lines (henceforth known as NtFX-KO) were obtained from two rounds of transformations of 300 *N. tabacum* cv. SR-1 explants. The formation of roots seemed to be slightly hampered and transformants initially displayed a reduced growth rate. However, they might be residual effects of the stable transformation and regeneration process, as we also occasionally see this phenotype when generating stable plant lines for recombinant protein production (van Dolleweerd et al., 2014). In subsequent seed-propagated generations, NtFX-KO transformants matured at similar times compared to wild-type plants. Knockout of *XylT* and *FucT* were screened by Western blotting of total soluble protein of crude leaf extracts using antibodies recognizing β(1,2)-xylose and α(1,3)-fucose (Figure 1). Several lines showed a reduction of β(1,2)-xylose and α(1,3)-fucose on *N*-glycans of plant proteins, while line #6 was devoid of β(1,2)-xylose and only displayed a faint signal for α(1,3)-fucose (Figure 1A). This line was selected and allowed to self-fertilize. A total of 9 plants from the T_1_ generation were grown and analyzed using the same immunoblotting assay. Eight out of nine T_1_ lines were completely devoid of β(1,2)-xylose and α(1,3)-fucose, indicating gene inactivation (Figure 1B).

### Transient expression and glycan analysis of the antibody VRC01 in selected NtFX-KO candidates

**Figure 2:**
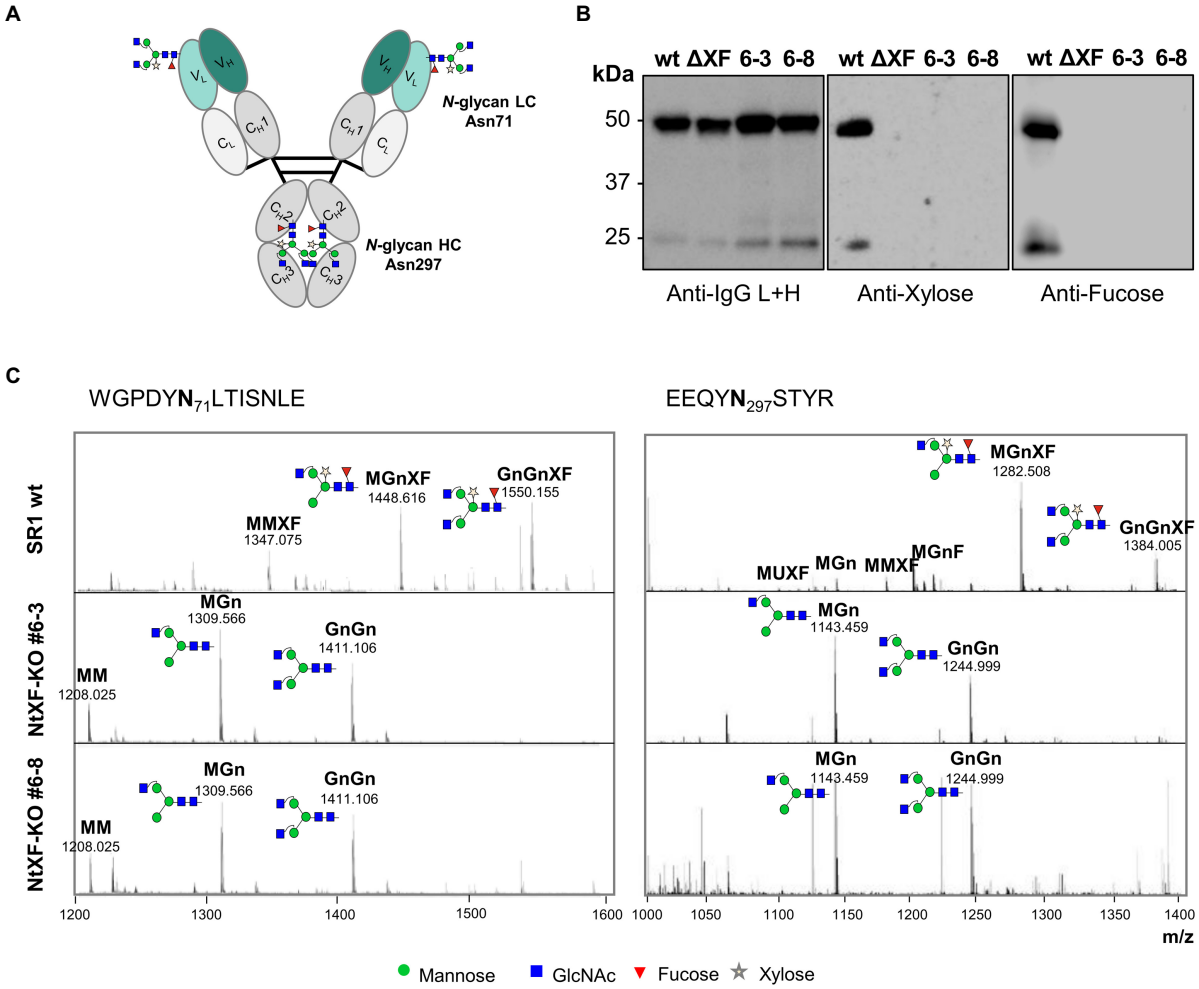
Analysis of *N*-glycosylation of purified VRC01 from transgenic NtFX-KO lines. **(A)** *N-*linked glycosylation sites on VRC01. There are four sites in total – two at position Asn71 in the FR3 of the light chain V_L_ region, and two at position Asn297 of the heavy chain CH2 region. **(B)** Western blot analysis of 200 ng of VRC01 purified from *Nicotiana tabacum* wild-type (wt), transgenic lines NtFX-KO #6–3 and #6–8, as well as *N. benthamiana* ΔXT/FT (ΔXF; kindly provided by BOKU Vienna), under reducing conditions. Membranes were probed with HRP-labeled anti-IgG L + H chain antisera as well as rabbit anti-xylose and anti-fucose antisera followed by HRP-labeled goat anti-rabbit IgG antisera. **(C)** Site-specific *N*-glycosylation of purified mAbs analyzed by LC-ESI-MS of the light-chain (WGPDYNLTISNLE) and heavy-chain (EEQYNSTYR) glycopeptides [both (M + 2H)^2+^]. N-glycans were abbreviated according to the ProGlycAn system (www.proglycan.com). Please note that just one possible isomer was shown. The symbols for the monosaccharides were drawn according to the nomenclature from the Consortium for Functional Glycomics.

The suitability of the NtFX-KO transformants for the production of recombinant proteins lacking plant-specific *N*-glycan modifications was tested by transient expression of an anti-HIV-1 hIgG1 antibody VRC01, which, apart from the *N*-linked glycosylation at position Asn297 in the CH2 domain, carries an additional *N*-glycan in the FR3 of the light chain V_L_ region at Asn71 (Figure 2A, Teh et al., 2014). Purification yields of the VRC01 antibody from vacuum-infiltrated detached leaves of NtFX-KO lines (#6–3: 9.1 ± 1.0 mg/kg LFW; #6–8: 6.2 ± 0.1 mg/kg LFW) were similar to wild-type plants (11.1 ± 0.8 mg/kg LFW). Immunoblotting using anti-β(1,2)-xylose and anti-α(1,3)-fucose antibodies produced strong bands for wild-type VRC01 heavy and light chains at 50 kDa and 25 kDa respectively, showing the presence of plant-specific fucose and xylose at both glycosylation sites (Figure 2B). In contrast, those signals were not detected for VRC01 produced in the NtFX-KO lines indicating the absence of these plant-specific *N*-glycan modifications.

Site-specific *N*-glycan analysis by LC-ESI-MS of VRC01 purified from wild-type and putative NtFX-KO transformants was performed for a more detailed identification of the attached glycosylation (Figure 2C). The relative abundance of distinct glycoforms at both *N*-glycosylation sites was quantified by integration of identified peaks of the obtained mass spectra (Table 1). The CH2 domain and light-chain *N*-glycosylation sites of VRC01 purified from wild-type as well as NtFX-KO plants #6–3 and #6–8 were almost fully occupied (Table 1). The mass-spectra of wild-type VRC01 displayed very homogenous *N*-glycosylation profiles at both sites with complex-type core-fucose and -xylose containing glycans (GnGnXF and MGnXF) typically observed in plants. On the other hand, the *N*-glycosylation profiles in the NtFX-KO plants #6–3 and #6–8 displayed GnGn/MGn as major glycoforms with a complete absence of plant-specific modifications at both *N*-glycosylation sites (Figure 2C; Table 1). This is encouraging as previous attempts using the pFGC-FucT construct only reduced fucosylation in the CH2 but not the light-chain resident *N*-glycan site (Supplementary Tables S3, S4). This strongly indicated complete gene knockout of the respective glycosyltransferases in lines NtFX-KO #6–3 and #6–8.

**Table 1:** Quantification of the relative abundance of *N*-glycans detected on the light (N71) and heavy chains (N297) of VRC01 hIgG1 produced in *Nicotiana tabacum* cv. SR-1 wild-type and NtFX-KO lines.

Abbreviation	Structure	Lines					
		wt		NtFX-KO #6–3		NtFX-KO #6–8	
		N71	N297	N71	N297	N71	N297
Non-glycosylated		0.0%	3.2%	0.0%	2.8%	0.0%	5.9%
GnGnXF	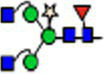	36.9%	16.1%	0.0%	0.0%	0.0%	0.0%
MGnXF	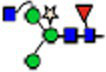	39.4%	48.4%	0.0%	0.0%	0.0%	0.0%
MMXF	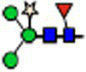	15.3%	5.9%	0.0%	0.0%	0.0%	0.0%
MUXF	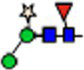	3.6%	1.0%	0.0%	0.0%	0.0%	0.0%
AGnF	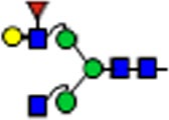	4.8%	0.0%	0.5%	0.0%	1.4%	0.0%
GnGnF	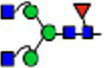	0.0%	3.1%	0.0%	0.0%	0.0%	0.0%
MGnF	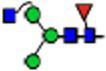	0.0%	7.8%	0.0%	0.0%	0.0%	0.0%
MMF	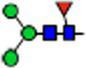	0.0%	1.2%	0.0%	0.0%	0.0%	0.0%
GnGn	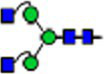	0.0%	3.4%	34.7%	38.1%	36.5%	43.5%
MGn	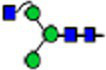	0.0%	4.5%	43.8%	53.6%	38.0%	50.7%
MM	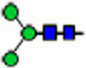	0.0%	1.0%	15.7%	3.0%	18.5%	0.0%
MU	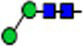	0.0%	0.0%	4.4%	0.0%	4.6%	0.0%
Man7	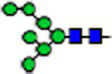	0.0%	3.2%	0.4%	1.7%	0.5%	0.0%
Man8	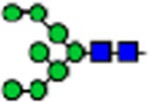	0.0%	0.9%	0.3%	0.8%	0.5%	0.0%
Man9	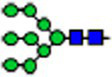	0.0%	0.3%	0.2%	0.0%	0.0%	0.0%

*N*-glycans were abbreviated according to the ProGlycAn system (www.proglycan.com). The symbols for the monosaccharides were drawn according to the nomenclature from the Consortium for Functional Glycomics.

The presence of *N*-glycan structures with a single terminal GlcNAc and paucimannosidic glycans in both *N*-glycosylation sites of wild-type VRC01 (GnMXF/MGnXF/MMXF) and in a lesser extent in NtFX-KO VRC01 (GnM/MGn/MM) is often seen in plant-produced IgGs due to incomplete processing by *N*-acetylglucosaminyltransferase II (Dicker et al., 2016) or β-hexosaminidases activity in the post-Golgi compartment (Shin et al., 2017). Apart from the major glycoforms that are visible at the presented scale of the mass spectra, minor amounts of other glycoforms such as oligomannosidic glycans (Man7-9, up to 3%) at Asn297 and α(1,4)-fucose containing Le^a^ epitope at Asn71 (AGnF, 5% in wild-type VRC01, 0.5%–1.5% in NtFX-KO VRC01) were detected (Table 1). Le^a^ epitopes are not usually found in the CH2 resident *N*-glycan on human IgGs nor plant-made recombinant IgGs. However, it can sometimes be found in *N*-glycans attached to the variable regions of some IgGs, most probably due to α1,4-fucosyltransferase activity (Fitchette-Lainé et al., 1997; Léonard et al., 2002).

### Genomic analysis of NtFX-KO lines

**Figure 3:**
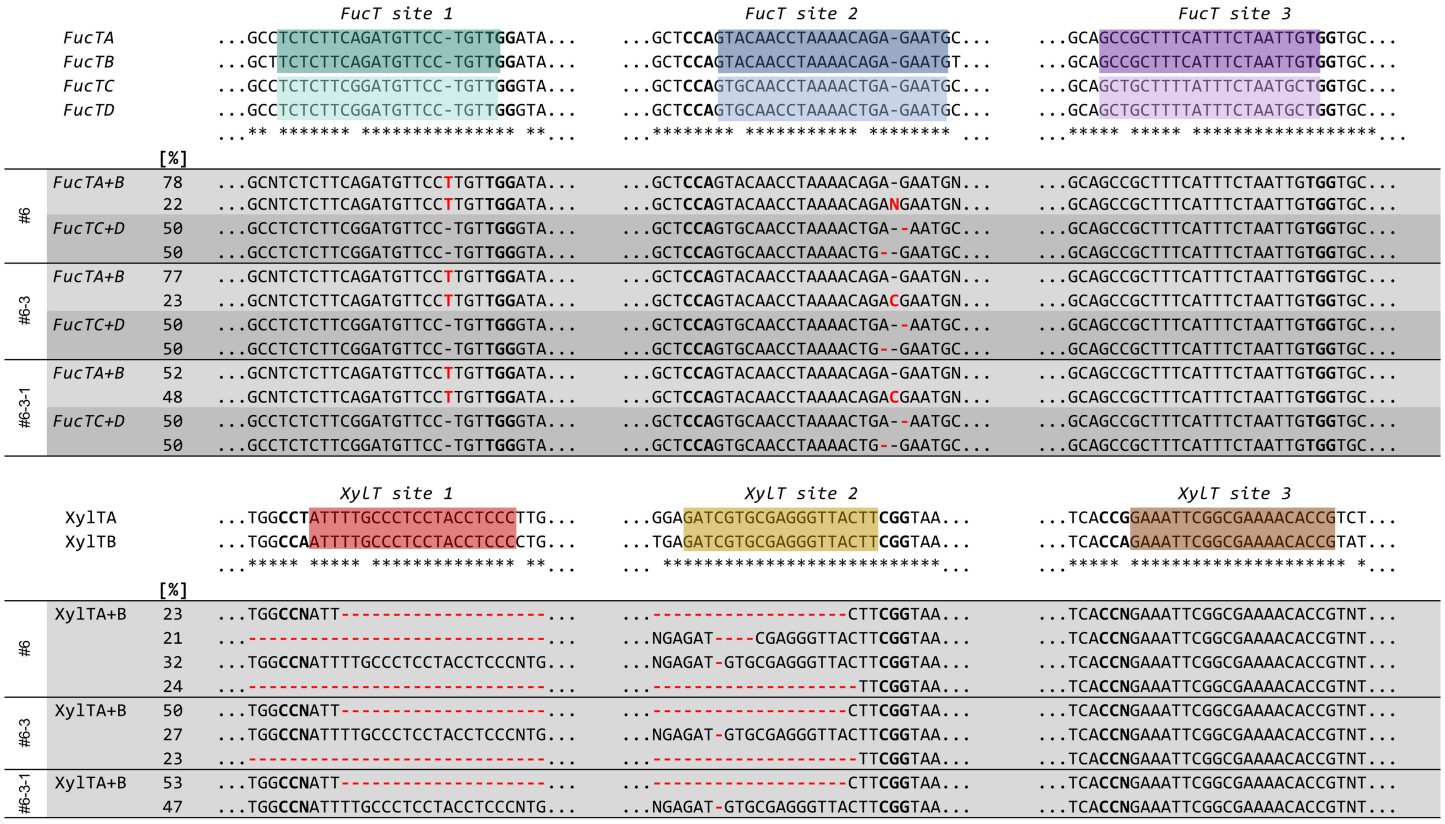
Genetic characterization of transgenic NtFX-KO line. Sequences of the regions targeted by pFGC-LFX in the *FucTA-D* and *XylTA-B* genes of NtFX-KO lines #6 (T_0_ generation), #6-3 (T_1_ generation), and #6-3-1 (T_2_ generation) were identified by Sanger sequencing, and the PCR amplicons analyzed using Synthego ICE. The corresponding wild-type sequences are shown above, the sgRNA target sequences are indicated by colored boxes, and the PAM sequences shown in bold.

To investigate the introduced mutations in the *FucT* and *XylT* loci of the NtFX-KO #6–3 line that was selected for further breeding, we have used PCR amplification of the targeted regions followed by Sanger sequencing and sequence analysis using the Synthego ICE analysis tool (Conant et al., 2022). Due to the high homology of the targeted regions in both β(1,2)-xylosyltransferase as well as the four α(1,3)-fucosyltransferase genes, primers simultaneously amplifying *FucTA/B, FucTC/D*, or *XylTA/B* had to be used (Supplementary Table S2). This resulted in overlapping sequencing traces that caused difficulties to differentiate the nature of introduced mutations in some instances, especially in earlier generations. Therefore, these are expressed as relative abundance for each targeted site in the FucTA/B, FucTC/D, and XylTA/B regions (%, Figure 3). Nonetheless, we identified mutations consisting mostly of +1 insertions or small deletions in at least one of the three target sites in all four *FucT* isotypes, and small deletions as well as fragment deletions in the *XylT* isotypes of T_0_ plants, with all reaching apparent homozygosity by the T_2_ generation. This demonstrated the high editing efficiency of the pFGC-LFX construct.

## Discussion

Ever since plants emerged as a promising manufacturing platform for recombinant protein therapeutics, concerns were raised that the *N*-glycans produced in plants could influence the functionality and immunogenicity of plant-produced glycoproteins (van Beers and Bardor, 2012). Consequently, efforts have been made to modify the *N*-glycosylation pathway in a variety of plant species using different strategies such as RNAi (Strasser et al., 2008), and more recently targeted nucleases (Li et al., 2016; Hanania et al., 2017; Mercx et al., 2017). Here, we exploited the efficiency and multiplexing capability of the CRISPR/Cas9 system to knockout two β(1,2)-xylosyltransferase and four α(1,3)-fucosyltransferase genes in *Nicotiana tabacum* cv. SR-1 using a construct that has already been successfully used in the *N. tabacum* BY-2 cell line. We established NtFX-KO lines, confirmed the absence of each glycosyltransferase activity by analysing both the *N*-glycans of total soluble proteins as well as the *N*-glycans of the recombinant anti-HIV-1 antibody VRC01 transiently expressed in these plant lines. VRC01 was selected as a model antibody for screening of putative knockout lines because *N*-glycan sites were present in both the CH2 region of the heavy chain, as well as the FR3 region of the light chain. We also confirmed all four *XylT* and eight *FucT* targeted alleles were knocked out at DNA level. To our knowledge, this is the first report of a complete knockout of *FucT* and *XylT* activity in *N. tabacum* plants.

Initially, several in-house CRISPR/Cas9 constructs targeting different regions of *FucT* and *XylT* isotypes were screened and achieved editing efficiencies of up to 90% and 30% at T_2_ and T_3_, respectively. However, these did not generate fully functional knockout lines of the respective glycosyltransferases. Western blot showed that no complete *XylT* knockout lines have been generated using the in house pFGC-XylT construct. On the other hand, there were putative *FucT* knockout lines generated by the pFGC-FucT construct that had undetectable signal in the anti-α(1,3)-fucose Western blot. However, site-specific mass spectrometric *N*-glycan analysis of the anti-HIV-1 antibody VRC01 produced in these lines showed that while fucosylation of the CH2 resident *N*-glycan was significantly reduced, the light-chain *N*-glycan was virtually unaffected. The light-chain *N-*glycan is more exposed and hence might be more accessible for core-fucosylation (Thaysen-Andersen and Packer, 2012; Göritzer et al., 2017).

Using the alternative pFGC-LFX construct, we managed to generate the NtFX-KO lines. Site-specific *N*-glycosylation analysis of VRC01 produced in these lines revealed that *N*-glycans of both the light-chain and CH2 domain display a very homogenous glycosylation profile with GnGn and MGn as major glycoforms (>95%) in the knockout lines without the presence of plant-specific modifications. Furthermore, paucimannosidic *N*-glycans are reduced in these KO lines (50%) compared to wild-type plants (70%). This correlated with a recent study that demonstrated that in *N. benthamiana,* the presence of the core α(1,3)-fucose on *N*-glycans enhances the trimming of GlcNAc residues by β-hexosaminidases located in the plasma membrane of leaf epidermal cells (Shin et al., 2017).

The pFGC-FucT/pFGC-XylT and the pFGC-LFX constructs targeted different regions of the *FucT* and *XylT* genes. pFGC-FucT or pFGC-XylT targeted exons 2 and 1 of the respective genes. On the other hand, pFGC-LFX targeted exon 3 of *FucT,* and a different region of the *XylT* exon 1. Furthermore, due to the absence of a complete annotated *N. tabacum* genome at the beginning of the study (the draft annotated genome of *N. tabacum* cv. K326 was only available recently), different strategies have been used to obtain the gene sequences to design the sgRNA. For pFGC-FucT and pFGC-XylT, *FucT* and *XylT* gene sequences have been obtained by using *N. benthamiana* orthologs as a template. The regions targeted by the sgRNAs were also chosen based on their similarity to a construct that successfully generated an *N. benthamiana FucT/XylT* knockout line (Jansing et al., 2019). On the other hand, the sgRNAs of pFGC-LFX were designed using *FucT* and *XylT* gene sequences from *N. tomentosiformis* and *N. sylvestris*, the parental lines of BY2 cells (Mercx et al., 2017). These reasons might explain their relative success rates in generating complete knockout lines.

Ma et al. (2015) had shown that a single vaginal administration of plant-made 2G12, another anti-HIV-1 antibody, to healthy females was well tolerated. This recombinant 2G12 was produced in *N. tabacum* cv. SR-1 with wild-type glycosylation. On the other hand, although there is no safety data available on infused plant-produced recombinant antibodies, ELELYSO^®^, currently in clinical use for treatment of Gaucher’s disease, is administered every other week at 60 U/kg (equivalent to 1.8 mg/kg; Pfizer New Zealand Limited, 2019). The main active ingredient of ELELYSO^®^ (Taliglucerase alfa) is produced in carrot cells and has wild-type plant glycans. In comparison, two or four 30 mg/kg infusions of anti-HIV-1 antibody 3BNC117 was given every three or 2 weeks for viral suppression after interruption of antiretroviral therapy (Scheid et al., 2016). It is still not clear whether infusions of recombinant proteins containing plant-specific glycans at a higher concentration might cause an immunogenic reaction.

The elimination of plant-specific modifications is not only desirable for regulatory caution – the homogeneity of glycosylation in plants compared to proteins made in mammalian platforms is also an advantage. Therapeutic monoclonal antibodies devoid of core-fucose have significantly improved Fc mediated effector functions including ADCC (Zeitlin et al., 2011; Stelter et al., 2020). Furthermore, these lines can be used as a starting point for further glycoengineering to produce human-like *N*-glycosylation patterns, such as the introduction of pathways for the production, activation, transport and transfer of sialic acid to terminal galactose residues which confer different properties to monoclonal antibodies such as extended half-life (Castilho et al., 2010).

Knockout of the eight *FucT* and four *XylT* alleles in line NtFX-KO #6–8 was further investigated at DNA level. Editing was very efficient, with mutations in all *FucT* and *XylT* alleles in the T_0_ generation and homozygosity reached by generation T_2_. Given that the genome of *N. tabacum* cv. SR-1 was not fully sequenced nor annotated, unknown *FucT* or *XylT* genes that were not targeted by the CRISPR-Cas9 constructs cannot be ruled out. However, in this case, any activity in the NtFX-KO lines must be below detection limits since Western blotting of total soluble protein as well as mass spectrometric analysis of recombinantly produced VRC01 did not show any residual signal for β(1,2)-xylose or core α(1,3)-fucose. The efficiency of targeting can be explained by the fact that Cas9 and the sgRNAs were stably integrated into the genome and continuously expressed. In the future, the Cas9 construct can be eliminated from the genome of the transformed plants by Mendelian segregation, which would be monitored for subsequent generations. A drawback of the CRISPR/Cas9 editing system is that, although rare, off-targets might be generated (Puchta, 2017). An exhaustive investigation will be time consuming but essential phenotypes were confirmed. Regeneration and initial growth of the transformants at T_0_ was reduced. However, this was not observed in subsequent generations. The absence of an obvious phenotype in flowering, seed set, or specific recombinant protein expression and secretion, suggested that the presence of the plant-specific xylose and core-fucose residues is not critical for a molecular farming host.

In conclusion, we have demonstrated the generation of transgenic *Nicotiana tabacum* cv. SR-1 plants with functional knockouts of six genes responsible for non-human glycan structures. We have showed that these NtFX-KO transgenic lines can express the broadly neutralizing anti-HIV-1 antibody VRC01 that are completely devoid of β(1,2)-xylose and α(1,3)-fucose residues, all without significant loss of yield compared to wild-type *N. tabacum* cv. SR-1. These data represent an important step toward humanizing the glycosylation of pharmaceutical proteins in *Nicotiana tabacum.*

## Data availability statement

The original contributions presented in the study are included in the article/Supplementary material, further inquiries can be directed to the corresponding author.

## Author contributions

KG, MG, and AT performed the experiments and analyzed the data. SM and CN developed the pFGC-LFX CRISPR/Cas9 construct. CG and RF performed the mass spectrometry analysis. AT and JM conceived and supervised the project. KG wrote the first draft of the manuscript. All authors contributed to the article and approved the submitted version.

## Funding

We would like to thank the generous support from the European Research Council’s Horizon 2020 programme under Grant Agreements 760331 (Newcotiana) and 774078 (Pharma-Factory), as well as the Sir Joseph Hotung Charitable Trust. KG would also like to acknowledge the support of the Austrian Science Fund Erwin Schrödinger Fellowship J-4583.

## Conflict of interest

The authors declare that the research was conducted in the absence of any commercial or financial relationships that could be construed as a potential conflict of interest.

## Publisher’s note

All claims expressed in this article are solely those of the authors and do not necessarily represent those of their affiliated organizations, or those of the publisher, the editors and the reviewers. Any product that may be evaluated in this article, or claim that may be made by its manufacturer, is not guaranteed or endorsed by the publisher.
