# Supplementary material for: Engineering the N-glycosylation pathway of Nicotiana tabacum for molecular pharming using CRISPR/Cas9
Source: Front Plant Sci. 2022 Sep 8;13:1003065. doi: 10.3389/fpls.2022.1003065 (PMC9493077; doi:10.3389/fpls.2022.1003065)
Supplement: Supplementary file 1 [file Data_Sheet_1.docx]

**Supplementary Table S1: Cutting efficiency of individual sgRNAs targeting *FucT* (F1-F12) or *XylT* (X1-X6) as determined by Indel detection by amplicon analysis (IDAA).** sgRNAs chosen for multiplexing in the pFGC-FucT and pFGC-XylT constructs are highlighted and in bold. The cutting efficiencies might be overestimates, but relative percentages were considered to select the sgRNAs for stable transformation.

| **sgRNA** | **% WT** | **% Indel** |
| --- | --- | --- |
| F1 | 42.5 | 57.5 |
| **F2** | 23 | 77 |
| F3 | 78 | 22 |
| F4 | 80 | 20 |
| **F5** | 0 | 100 |
| **F6** | 20 | 80 |
| **F7** | 36 | 64 |
| F8 | 91 | 9 |
| F10 | 30 | 70 |
| **F11** | 21.5 | 78.5 |
| F12 | 80 | 20 |
| X1 | 100 | 0 |
| **X2** | 82 | 18 |
| X3 | 100 | 0 |
| **X4** | 0 | 100 |
| **X5** | 63 | 37 |
| **X6** | 0 | 70 |

**Supplementary Table S2:** Primers used for amplification of *FucT* and *XylT* genes regions of *N. tabacum* cv. SR-1 targeted by the pFGC-LFX CRISPR/Cas9 construct.

| **Genes** | **Forward primer** | **Reverse primer** |
| --- | --- | --- |
| *FucTA, FucTB, FucTC’ FucTD* | 5’-GCACGACGAAGGGGATATGAT-3’ | 5’-TGATATTTGCCCTTTCAAGG-3’ |
| *FucTA, FucTB* | 5’-ACATTACTTTGGCACGACGAA-3’ | 5’-TACCGCTTCAGTGCTTCCAC-3’ |
| *FucTC, FucTD* | 5’-CACGACGAAGGGGATATGAT-3’ | 5’-GTCCACATTTCCATCCCGGT-3’ |
| *XylTA, XylTB* | 5’-ATCACTTCCGTCACAAATCCC-3’ | 5’-ACCGACTCCAATTTCTCGCC-3’ |

**Supplementary Table S3:** Quantification of the relative abundance (%) of *N*-glycans detected on the heavy chains of VRC01 hIgG1 produced in *N. tabacum* SR-1 wild-type (wt) and the T_2_ generation of putative *FucT* knockout line 7-21-1 generated using the pFGC-FucT construct. *N*-glycans were abbreviated according to the ProGlycAn system ([www.proglycan.com](http://www.proglycan.com)).

|  | | **T2 7-21-1** | | **wt** |
| --- | --- | --- | --- | --- |
| Man8 | | 0.00 | | 1.16 |
| Man9 |  | |  |  |
| MMF | | 0.60 | | 0.53 |
| MMX | | 0.52 | |  |
| MGnF | | 0.69 | | 0.74 |
| MGnX | | 1.54 | | 0.73 |
| GnGnXF | | 19.32 | | 71.45 |
| GnGnF | | 4.67 | | 6.19 |
| GnGnX | | 31.92 | | 2.50 |
| GnGn | | 27.93 | | 1.22 |
| MGnXF | | 3.55 | | 5.73 |
| Man5 | | 1.50 | | 0.96 |
| Man6 | | 0.65 | | 1.01 |
| not glyc. | | 3.08 | |  |
| MMXF | | 2.90 | | 5.77 |
| Man7 | | 1.13 | | 2.02 |
| GnM | | 1.60 | |  |

**Supplementary Table S4:** Quantification of the relative abundance (%) of *N*-glycans detected on the light chains of VRC01 hIgG1 produced in *N. tabacum* cv. SR-1 wild-type (wt) and the T_2_ generation of putative *FucT* knockout line 7-21-1 generated using the pFGC-FucT construct. *N*-glycans were abbreviated according to the ProGlycAn system ([www.proglycan.com](http://www.proglycan.com)).

|  | **T2 7-21-1** | **wt** |
| --- | --- | --- |
| MM |  |  |
| MMF |  |  |
| MMX |  |  |
| MGnF |  |  |
| MGnX | 3.12 | 3.01 |
| GnGnXF | 62.10 | 58.52 |
| GnGnF |  |  |
| GnGnX | 14.70 | 1.01 |
| GnGn |  |  |
| MGnXF | 12.03 | 7.95 |
| MGn |  |  |
| not glyc. |  |  |
| MMXF | 7.99 | 29.50 |

**N. Tab FucT mRNA alignment**

**FucT for 1 F1>**

gi|1025362229|ref|XM_016657530.1| ------------------------------------------------------ATGTCA

gi|1025193416|ref|XM_016620229.1| ---------------------------------------------ATGAGATCTTCGTCA

gi|1027852219|ref|NM_001324945.1| **ATGGCAACAGTTATTCCAA**TTCAAAGGTTACCAAGATTTGAAGGTGTTGGGTCATCATCA

gi|297306777|dbj|AB498916.1| **ATGGCAACAGTTATTCCAA**TTCAAAGGTTACCAAGATTTGAAGGTGTTGGGTCATCATCA

gi|1025416688|ref|XM_016585847.1| **ATGGCAACAGTTGTGCCAA**TTCAAAGATTACCAAGATTTGAAGGTGTTGGGTCATCATCA

**FucT for a <F2**

gi|1025362229|ref|XM_016657530.1| AATTCAA**ACGCACCCAATAAACAATGG**CGCAATTGGTTGCCTCTCTTCGTTGCCCTAGTG

gi|1025193416|ref|XM_016620229.1| AATTCAA**ACGCACCCAATAAACAATGG**CGCAATTGGTTGCCTCTGTTCTTTGCCCTAGTG

gi|1027852219|ref|NM_001324945.1| CCTACAAACGTTCCCCTTAAGAAATGGTCCAATTGGCTACCTCTAGTAGTTGCACTTGTG

gi|297306777|dbj|AB498916.1| CCTACAAACGTTCCCCTTAAGAAATGGTCCAATTGGCTACCTCTAGTAGTTGCACTTGTG

gi|1025416688|ref|XM_016585847.1| CCTACAAATGTTCCCCAAAAGAAATGGTCCAATTGGCTACCTCTAGTAGTTGCACTTGTG

**G**

**<F7**

gi|1025362229|ref|XM_016657530.1| GTTATAGCAGAGTTTTCTTTTCTGGTTCGACTCGACGTGGCTGAAAAAGCCA--------

gi|1025193416|ref|XM_016620229.1| GTTATAGCAGAGATTTCTTTTCTGGTTCGACTCGACGTGGCTGAAAAAGCCA--------

gi|1027852219|ref|NM_001324945.1| GTTATAGTTGAAATTACATTTCTGGGT**CGACTGGACATGGCTGAA**AAAGCCAACCTGGTC

gi|297306777|dbj|AB498916.1| GTTATAGTTGAAATTACATTTCTGGGT**CGACTGGACATGGCTGAA**AAAGCCAACCTGGTC

gi|1025416688|ref|XM_016585847.1| GTTATAGTTGAAATTGCATTTCTGGGT**CGACTAGACATGGCTGAA**AAAGCCAACCTAGTC

**FucT for 2**

**<F3**

gi|1025362229|ref|XM_016657530.1| -ACTCTTGGGCCGACTCGTTTTATCAGTTCACCACGGCGTCTTGGTCCACCTCTAAACTG

gi|1025193416|ref|XM_016620229.1| -ACTCTTGGGCCGACTCGTTTTATCAGTTCACCACAGCCTCTTGGTCCACCTCTAAACTG

gi|1027852219|ref|NM_001324945.1| AAC**TCTTGGACTGACTCATTTTAC**CAGTTTACGACGTCGTCTTGGTCAACCTCCAAAGTG

gi|297306777|dbj|AB498916.1| AAC**TCTTGGACTGACTCATTTTAC**CAGTTTACGACGTCGTCTTGGTCAACCTCCAAAGTG

gi|1025416688|ref|XM_016585847.1| AAC**TCTTGGACTGACTCATTTTAC**CAGTTTACGACGTCGTCTTGGTCAACCTCCAAAGTG

**FucT rev 1**

F**8>**

gi|1025362229|ref|XM_016657530.1| TCTGCTGACCACGGCGACGTTGAGGAGGTCCAGTTGGGTGTTTTGAGTGGTGAGTTATTG

gi|1025193416|ref|XM_016620229.1| GCTGTTGACCACGGCGACGTTGAGGAGGTCCAGTTGGGTATT---T------------TG

gi|1027852219|ref|NM_001324945.1| GAAATTAGT----GAGAC-----------TGGGTTGGGTGTGTTGA------------GG

gi|297306777|dbj|AB498916.1| GAAATTAGT----GAGAC-----------TGGGTTGGGTGTGTTGA------------GG

gi|1025416688|ref|XM_016585847.1| GAAATTAAT----GAGGC-----------TGGGTTGGCTGTGTTAA------------GG

**Supplementary Figure S1.** Alignment of *N. tabacum* α(1,3)-Fucosyltransferase (*FucT*) mRNA, the location of protospacer elements and PCR amplification primers. Protospacer elements are in boxes with arrows showing their direction (< for sense and > for antisense). The protospacer elements are in the format of GN_20_-GG with the first ‘G’ and the Protospacer Adjacent Motif (PAM) sequences NGG highlighted in yellow or grey. Primers for PCR amplification are in bold and underlined. Reverse primers are shown as their reverse-complement counterpart.

<F**12 F4>**

gi|1025362229|ref|XM_016657530.1| AGTGGTGACTTTGATCAGGGCTTCGTACCTGGGAGTTGCGAGGAATGGTTGGAAAAGGA**A**

gi|1025193416|ref|XM_016620229.1| AGTGGTGAGTTTGATCAGGGCTTCGTACCCGGGAGTTGCGAGGAGTGGTTGGAAAGGGA**A**

gi|1027852219|ref|NM_001324945.1| AGTAGTGAGGTTGATCGGAATTTGGAAACTGGGAGCTGTGAGGAGTGGTTGGAAAAGGAG

gi|297306777|dbj|AB498916.1| AGTAGTGAGGTTGATCGGAATTTGGAAACTGGGAGCTGTGAGGAGTGGTTGGAAAAGGAG

gi|1025416688|ref|XM_016585847.1| AGTGGTGAGATTGATCGGAATTTGGAAACTGGGAGCTGTGAGGAGTGGTTGGAAAGGGAG

**G**

**FucT rev a**

gi|1025362229|ref|XM_016657530.1| **GATTCTGTGGCTTATTCGAG**GGATTTTGATAATGAACCAATTTTTGTTCATGGGCCTGGA

gi|1025193416|ref|XM_016620229.1| **GATTCTGTGGCTTATTCGAG**GGATTTTGATAATGAACCAATTTTTGTTCATGGGCCTGGA

gi|1027852219|ref|NM_001324945.1| GATTCTGTGGAGTATTCTAGAGATTTTGACAAAGACCCAATTTTTG**TTCATGGCGGCGAA**

gi|297306777|dbj|AB498916.1| GATTCTGTGGAGTATTCTAGAGATTTTGACAAAGACCCAATTTTTG**TTCATGGCGGCGAA**

gi|1025416688|ref|XM_016585847.1| GATTCTGTGGAGTATTCTAGAGATTTTGACAAAGATCCAATTTTTG**TTCATGGCGGCGAA**

**FucT rev 2**

**Intron site FucT for b**

gi|1025362229|ref|XM_016657530.1| CAGGAATT**GAAATCCTGTTCCATAGGATG**TAAGTTTGGAACAGATTCCGATAAGAAGCCT

gi|1025193416|ref|XM_016620229.1| CAGGAATT**GAAAACCTGTTCCGTAGGATG**TAAGTTTGGAACAGATTCCGATAAGAAGCCT

gi|1027852219|ref|NM_001324945.1| **AAGG**ATTGG**AAGTCTTGTGCCGTAGGA**TGTAACTTTGGTGTGGATTCTGAAAAGAAGCCT

gi|297306777|dbj|AB498916.1| **AAGG**ATTGG**AAGTCTTGTGCCGTAGGA**TGTAACTTTGGTGTGGATTCTGAAAAGAAGCCT

gi|1025416688|ref|XM_016585847.1| **AAGG**ATTGG**AAGTCTTGTGCCGTAGGA**TGTAACTTTGGTGTGGATTCTGATAAGAAGCCT

**FucT for 3**

**F5>**

gi|1025362229|ref|XM_016657530.1| GATGCGGCATTTCGGCTACCACAACAAGCTGGCACAGCTAGTGTGCTACGGTCGATGGAG

gi|1025193416|ref|XM_016620229.1| GATGCAGCATTTCGGCTACCACAACAAGCTGGTACAGCTAGTGTGCTACGGTCAATGGAG

gi|1027852219|ref|NM_001324945.1| GATGCGGCATTTGGGACACCACAACAGGCTGGCACGGCTAGCGTGCTTCGGTCAATGGAG

gi|297306777|dbj|AB498916.1| GATGCGGCATTTGGGACACCACAACAGGCTGGCACGGCTAGCGTGCTTCGGTCAATGGAG

gi|1025416688|ref|XM_016585847.1| GATGCGGCATTTGGGACACCACAACAGGCTGGCACGGCTAGCGTGCTTCGGTCAATGGAG

**<F9** **FucT rev b*Intron site**

gi|1025362229|ref|XM_016657530.1| TCAGCTCAATACTATGCAGAGAACAACATTACTTTGGC**ACGACGAA**GGGGATATGATGTT

gi|1025193416|ref|XM_016620229.1| TCAGCTCAATACTATGCAGAGAACAACATTACTTTGGC**ACGACGAA**GGGGATATGATGTT

gi|1027852219|ref|NM_001324945.1| TCAGCTCAATACTATCCTGAGAAC**AACATCGTTATGGCACGACGA**AGGGGATATGATATT

gi|297306777|dbj|AB498916.1| TCAGCTCAATACTATCCTGAGAAC**AACATCGTTATGGCACGACGA**AGGGGATATGATATT

gi|1025416688|ref|XM_016585847.1| TCTGCTCAATACTATCCTGAGAAC**AACATCATTACCGCACGACGA**AGGGGATATGATATT

**FucT rev 3**

**Supplementary Figure S1 (continued).** Alignment of *N. tabacum* α(1,3)-Fucosyltransferase (*FucT*) mRNA, the location of protospacer elements and PCR amplification primers. Protospacer elements are in boxes with arrows showing their direction (< for sense and > for antisense). The protospacer elements are in the format of GN_20_-GG with the first ‘G’ and the Protospacer Adjacent Motif (PAM) sequences NGG highlighted in yellow or grey. Primers for PCR amplification are in bold and underlined. Reverse primers are shown as their reverse-complement counterpart.

**FucT for c**

gi|1025362229|ref|XM_016657530.1| GTAATGACAACAAGCCTCTCTTCAGATGTTCCTGTTGGATATTTCTCTTGGGCTGA**GTAT**

gi|1025193416|ref|XM_016620229.1| GTAATGACAACAAGCTTCTCTTCAGATGTTCCTGTTGGATACTTCTCTTGGGCTGA**GTAT**

gi|1027852219|ref|NM_001324945.1| GTAATGACAACAAGCCTCTCTTCGGATGTTCCTGTTGGGTACTTCTCTTGGGCGGAGTAT

gi|297306777|dbj|AB498916.1| GTAATGACAACAAGCCTCTCTTCGGATGTTCCTGTTGGGTACTTCTCTTGGGCGGAGTAT

gi|1025416688|ref|XM_016585847.1| GTAATGACAACAAGCCTCTCTTCGGATGTTCCTGTTGGGTACTTCTCTTGGGCGGAGTAC

gi|1025362229|ref|XM_016657530.1| **GATATCATGGCTCCAG**TACAACCTAAAACAGAGAATGCCTTAGCAGCCGCTTTCATTTCT

gi|1025193416|ref|XM_016620229.1| **GATATCATGGCTCCAG**TACAACCTAAAACAGAGAATGTCTTAGCAGCCGCTTTCATTTCT

gi|1027852219|ref|NM_001324945.1| GATATAATGGCTCCAGTGCAACCTAAAACTGAGAATGCGTTAGCAGCTGCTTTTATTTCT

gi|297306777|dbj|AB498916.1| GATATAATGGCTCCAGTGCAACCTAAAACTGAGAATGCGTTAGCAGCTGCTTTTATTTCT

gi|1025416688|ref|XM_016585847.1| GATATAATGGCTCCAGTGCAACCTAAAACTGAGAATGCATTAGCAGCTGCTTTTATTTCT

**F10>**

gi|1025362229|ref|XM_016657530.1| AATTGTGGTGCTCGCAACTTCCGCTTGCAAGCTTTAGAAGCCCTTGAAAGGGCAAATATC

gi|1025193416|ref|XM_016620229.1| AATTGTGGTGCTCGCAATTTCCGCTTGCAAGCTTTAGAAGCCCTTGAAAGGGCAAATATC

gi|1027852219|ref|NM_001324945.1| AATTGTGGTGCTCGCAACTTCCGGTTACAGGCTCTTGAAGTCCTTGAAAGGGCAAATATC

gi|297306777|dbj|AB498916.1| AATTGTGGTGCTCGCAACTTCCGGTTACAGGCTCTTGAAGTCCTTGAAAGGGCAAATATC

gi|1025416688|ref|XM_016585847.1| AATTGTGGTGCTCGCAACTTCCGGTTGCAGGCTCTTGAAGTCCTTGAAAGGGCAAATATC

**<F11**

gi|1025362229|ref|XM_016657530.1| AGAATTGATTCTTATGGCAGTTGTCATCATAACAGGGATGGAAGAGTTGACAAAGTGGAA

gi|1025193416|ref|XM_016620229.1| AGAATTGATTCTTATGGCAGTTGTCATCATAACAGGGATGGAAGAGTTGACAAAGTGGAA

gi|1027852219|ref|NM_001324945.1| AAGATTGATTCTTTTGGCAGTTGTCATCGTAACCGGGATGGAAATGTGGACAAAGTGGAA

gi|297306777|dbj|AB498916.1| AAGATTGATTCTTTTGGCAGTTGTCATCGTAACCGGGATGGAAATGTGGACAAAGTGGAA

gi|1025416688|ref|XM_016585847.1| AAGATTGATTCTTTTGGCAGTTGTCATCGTAACCGGGATGGAAATGTGGACAAAGTGGAA

**Supplementary Figure S1 (continued).** Alignment of *N. tabacum* α(1,3)-Fucosyltransferase (*FucT*) mRNA, the location of protospacer elements and PCR amplification primers. Protospacer elements are in boxes with arrows showing their direction (< for sense and > for antisense). The protospacer elements are in the format of GN_20_-GG with the first ‘G’ and the Protospacer Adjacent Motif (PAM) sequences NGG highlighted in yellow or grey. Primers for PCR amplification are in bold and underlined. Reverse primers are shown as their reverse-complement counterpart.

**<F6 (conserved motif)**

gi|1025362229|ref|XM_016657530.1| GCACTGAAGCGGTACAAGTTTAGCTTGGCTTTTGAGAATTCTAATGAGGAGGACTATGTA

gi|1025193416|ref|XM_016620229.1| GCACTGAAGCGGTACAAGTTTAGCTTGGCTTTTGAGAATTCTAATGAGGAGGACTATGTA

gi|1027852219|ref|NM_001324945.1| ACTCTCAAGCGCTATAAATTTAGCTTCGCTTTTGAGAATTCTAATGAGGAGGATTATGTC

gi|297306777|dbj|AB498916.1| ACTCTCAAGCGCTATAAATTTAGCTTCGCTTTTGAGAATTCTAATGAGGAGGATTATGTC

gi|1025416688|ref|XM_016585847.1| ACTCTCAAGCGCTACAAATTTAGCTTCGCTTTTGAGAATTCTAATGAGGAGGATTATGTC

**FucT rev c** **Putative intron site**

gi|1025362229|ref|XM_016657530.1| ACTGAAAAAT**TCTTTCAGTCTCTGGTAGC**TGGATCAATCCCTGTGGTGGTTGGTGCTCCA

gi|1025193416|ref|XM_016620229.1| ACTGAAAAAT**TCTTTCAGTCTCTGGTAGC**TGGATCAATCCCTGTGGTGGTTGGTGCTCCA

gi|1027852219|ref|NM_001324945.1| ACCGAAAAATTCTTCCAGTCTCTGGTAGCTGGATCAGTCCCTGTGGTGATTGGTGCTCCA

gi|297306777|dbj|AB498916.1| ACCGAAAAATTCTTCCAGTCTCTGGTAGCTGGATCAGTCCCTGTGGTGATTGGTGCTCCA

gi|1025416688|ref|XM_016585847.1| ACTGAAAAATTCTTCCAGTCCTTAGTAGCTGGATCAGTCCCCGTGGTGATTGGTGCTCCA . . .

**Supplementary Figure S1 (continued).** Alignment of *N. tabacum* α(1,3)-Fucosyltransferase (*FucT*) mRNA, the location of protospacer elements and PCR amplification primers. Protospacer elements are in boxes with arrows showing their direction (< for sense and > for antisense). The protospacer elements are in the format of GN_20_-GG with the first ‘G’ and the Protospacer Adjacent Motif (PAM) sequences NGG highlighted in yellow or grey. Primers for PCR amplification are in bold and underlined. Reverse primers are shown as their reverse-complement counterpart.

***N. tab* XylT mRNA alignment**

gi|77540264|gb|DQ192540.1| ATGAACAAGAAAAAGCTGAAATTTCTTGTTTCTCTCTTCGCTCTCAACTCAATCACTCTC

gi|58696563|emb|AJ627182.1| ATGAACAAGAAAAAGCTGAAATTTCTTGTTTCTCTCTTCGCTCTCAACTCAATCACTCTC

gi|1027858651|ref|NM_001325611.1| ATGAACAAGAAAAAGCTGAAATTTCTTGTTTCTCTCTTCGCTCTCAACTCAATCACTCTC

gi|1027859365|ref|NM_001324669.1| ATGAACAAGAAAAAGCTGAAATTTCTTGTTTCTCTCTTCGCTCTCAACTCAATCACTCTC

gi|72388788|gb|DQ119667.1| ATGAACAAGAAAAAGCTGAAATTTCTTGTTTCTCTCTTCGCTCTCAACTCAATCACTCTC

************************************************************

**XylT for a**

gi|77540264|gb|DQ192540.1| TATCTCTACTTCTCTTCCCACTCTG**ATCACTTCCGTCACAAATCC**CCCCAAAACCACTTT

gi|58696563|emb|AJ627182.1| TATCTCTACTTCTCTTCCCACTCTGATCACTTCCGTCACAAATCCCCCCAAAACCACTTT

gi|1027858651|ref|NM_001325611.1| TATCTCTACTTCTCTTCCCACTCTGATCACTTCCGTCACAAATCCCCCCAAAACCACTTT

gi|1027859365|ref|NM_001324669.1| TATCTCTACTTCTCTTCCCACTCTGATCACTTCCGTCACAAATCCCCCCAAAACCACTTT

gi|72388788|gb|DQ119667.1| TATCTCTACTTCTCTTCCCACTCTGATCACTTCCGTCACAAATCCCCCCAAAACCACTTT

************************************************************

gi|77540264|gb|DQ192540.1| CCTAATACCCAAAACCACTATTCCCTGTCGGAAAACCACCATGATAATTTCCACTCTTCT

gi|58696563|emb|AJ627182.1| CCTAATACCCAAAACCACTATTCCCTGTCGGAAAACCACCATGATAATTTCCACTCTTCT

gi|1027858651|ref|NM_001325611.1| CCTAATACCCAAAACCACTATTCCCTGTCGGAAAACCACCATGATAATTTCCACTCTTCT

gi|1027859365|ref|NM_001324669.1| CCTAATACCCAAAACCACTATTCCCTGTCGGAAAACCACCATGATAATTTCCACTCTTCT

gi|72388788|gb|DQ119667.1| CCTAATACCCAAAACCACTATTCCCTGTCGGAAAACCACCATGATAATTTCCACTCTTCT

************************************************************

X**1> X2>**

gi|77540264|gb|DQ192540.1| GTCACTTCCCAATATACCAAGCCTTGGCCAATTTTGCCCTCCTACCTCCCCTGGTCTCAG

gi|58696563|emb|AJ627182.1| GTCACTTCCCAATATACCAAGCCTTGGCCAATTTTGCCCTCCTACCTCCCCTGGTCTCAG

gi|1027858651|ref|NM_001325611.1| GTCACTTCCCAATATACCAAGCCTTGGCCAATTTTGCCCTCCTACCTCCCCTGGTCTCAG

gi|1027859365|ref|NM_001324669.1| GTCACTTCCCAATATACCAAGCCTTGGCCAATTTTGCCCTCCTACCTCCCCTGGTCTCAG

gi|72388788|gb|DQ119667.1| GTCACTTCCCAATATACCAAGCCTTGGCCAATTTTGCCCTCCTACCTCCCCTGGTCTCAG

************************************************************

**<X3** **XylT for b**

gi|77540264|gb|DQ192540.1| AATCCTAATGTTTCTTTGAGATCGTGCGAGGGTTACTTCGGTAATGGG**TTTACTCTCAAA**

gi|58696563|emb|AJ627182.1| AATCCTAATGTTTCTTTGAGATCGTGCGAGGGTTACTTCGGTAATGGGTTTACTCTCAAA

gi|1027858651|ref|NM_001325611.1| AATCCTAATGTTTCTTTGAGATCGTGCGAGGGTTACTTCGGTAATGGGTTTACTCTCAAA

gi|1027859365|ref|NM_001324669.1| AATCCTAATGTTTCTTTGAGATCGTGCGAGGGTTACTTCGGTAATGGGTTTACTCTCAAA

gi|72388788|gb|DQ119667.1| AATCCTAATGTTTCTTTGAGATCGTGCGAGGGTTACTTCGGTAATGGGTTTACTCTCAAA

************************************************************

**Supplementary Figure S2.** Alignment of *N. tabacum* β(1,2)-Xylosyltransferase (*XylT*) mRNA, the location of protospacer elements and PCR amplification primers. Protospacer elements are in boxes with arrows showing their direction (< for sense and > for antisense). The protospacer elements are in the format of GN_20_-GG with the first ‘G’ and the Protospacer Adjacent Motif (PAM) sequences NGG highlighted in yellow or grey. Primers for PCR amplification are in bold and underlined. Reverse primers are shown as their reverse-complement counterpart.

**XylT rev a**

gi|77540264|gb|DQ192540.1| **GTTGATCTTC**TCAAAAC**TTCGCCGGAGCTTCACCAGAA**ATTCGGCGAAAACACCGTATCC

gi|58696563|emb|AJ627182.1| GTTGATCTTCTCAAAACTTCGCCGGAGCTTCACCAGAAATTCGGCGAAAACACCGTATCC

gi|1027858651|ref|NM_001325611.1| GTTGATCTTCTCAAAACTTCGCCGGAGCTTCACCAGAAATTCGGCGAAAACACCGTATCC

gi|1027859365|ref|NM_001324669.1| GTTGATCTTCTCAAAACTTCGCCGGAGCTTCACCAGAAATTCGGCGAAAACACCGTATCC

gi|72388788|gb|DQ119667.1| GTTGATCTTCTCAAAACTTCGCCGGAGCTTCACCAGAAATTCGGCGAAAACACCGTATCC

************************************************************

**<X4**

gi|77540264|gb|DQ192540.1| GGCGACGGCGGATGGTTTAGGTGTTTTTTCAGTGAGACTTTGCAGAGTTCGATTTGCGAG

gi|58696563|emb|AJ627182.1| GGCGACGGCGGATGGTTTAGGTGTTTTTTCAGTGAGACTTTGCAGAGTTCGATTTGCGAG

gi|1027858651|ref|NM_001325611.1| GGCGACGGCGGATGGTTTAGGTGTTTTTTCAGTGAGACTTTGCAGAGTTCGATTTGCGAG

gi|1027859365|ref|NM_001324669.1| GGCGACGGCGGATGGTTTAGGTGTTTTTTCAGTGAGACTTTGCAGAGTTCGATTTGCGAG

gi|72388788|gb|DQ119667.1| GGCGACGGCGGATGGTTTAGGTGTTTTTTCAGTGAGACTTTGCAGAGTTCGATTTGCGAG

************************************************************

**<X5**

gi|77540264|gb|DQ192540.1| GGAGGTGCTATACGAATGAATCCGGACGAGATTTTGATGTCTCGTGGAGGCGAGAAATTG

gi|58696563|emb|AJ627182.1| GGAGGTGCTATACGAATGAATCCGGACGAGATTTTGATGTCTCGTGGAGGCGAGAAATTG

gi|1027858651|ref|NM_001325611.1| GGAGGTGCTATACGAATGAATCCGGACGAGATTTTGATGTCTCGTGGAGGCGAGAAATTG

gi|1027859365|ref|NM_001324669.1| GGAGGTGCTATACGAATGAATCCGGACGAGATTTTGATGTCTCGTGGAGGCGAGAAATTG

gi|72388788|gb|DQ119667.1| GGAGGTGCTATACGAATGAATCCGGACGAGATTTTGATGTCTCGTGGAGGCGAGAAATTG

************************************************************

**XylT for c**

gi|77540264|gb|DQ192540.1| GAGTCGGTTATTGGTAGGAGTGAAGATGATGAGCTGCCCGTGTTCAAAAAT**GGAGCTTTT**

gi|58696563|emb|AJ627182.1| GAGTCGGTTATTGGTAGGAGTGAAGATGATGAGCTGCCCGTGTTCAAAAATGGAGCTTTT

gi|1027858651|ref|NM_001325611.1| GAGTCGGTTATTGGTAGGAGTGAAGATGATGAGCTGCCCGTGTTCAAAAATGGAGCTTTT

gi|1027859365|ref|NM_001324669.1| GAGTCGGTTATTGGTAGGAGTGAAGATGATGAGCTGCCCGTGTTCAAAAATGGAGCTTTT

gi|72388788|gb|DQ119667.1| GAGTCGGTTATTGGTAGGAGTGAAGATGATGAGCTGCCCGTGTTCAAAAATGGAGCTTTT

************************************************************

**XylT rev b**

gi|77540264|gb|DQ192540.1| **CAGATTAAAGTT**ACT**GATAAACTGAAAATTGGGAAA**AAATTAGTGGATGAAAAAATCTTG

gi|58696563|emb|AJ627182.1| CAGATTAAAGTTACTGATAAACTGAAAATTGGGAAAAAATTAGTGGATGAAAAAATCTTG

gi|1027858651|ref|NM_001325611.1| CAGATTAAAGTTACTGATAAACTGAAAATTGGGAAAAAATTAGTGGATGAAAAAATCTTG

gi|1027859365|ref|NM_001324669.1| CAGATTAAAGTTACTGATAAACTGAAAATTGGGAAAAAATTAGTGGATGAAAAAATCTTG

gi|72388788|gb|DQ119667.1| CAGATTAAAGTTACTGATAAACTGAAAATTGGGAAAAAATTAGTGGATGAAAAAATCTTG

************************************************************

**Supplementary Figure S2 (continued).** Alignment of *N. tabacum* β(1,2)-Xylosyltransferase (*XylT*) mRNA, the location of protospacer elements and PCR amplification primers. Protospacer elements are in boxes with arrows showing their direction (< for sense and > for antisense). The protospacer elements are in the format of GN_20_-GG with the first ‘G’ and the Protospacer Adjacent Motif (PAM) sequences NGG highlighted in yellow or grey. Primers for PCR amplification are in bold and underlined. Reverse primers are shown as their reverse-complement counterpart.

**<X6**

gi|77540264|gb|DQ192540.1| AATAAATACTTACCGGAAGGTGCAATTTCAAGGCACACTATGCGTGAATTAATTGACTCT

gi|58696563|emb|AJ627182.1| AATAAATACTTACCGGAAGGTGCAATTTCAAGGCACACTATGCGTGAATTAATTGACTCT

gi|1027858651|ref|NM_001325611.1| AATAAATACTTACCGGAAGGTGCAATTTCAAGGCACACTATGCGTGAATTAATTGACTCT

gi|1027859365|ref|NM_001324669.1| AATAAATACTTACCGGAAGGTGCAATTTCAAGGCACACTATGCGTGAATTAATTGACTCT

gi|72388788|gb|DQ119667.1| AATAAATACTTACCGGAAGGTGCAATTTCAAGGCACACTATGCGTGAATTAATTGACTCT

************************************************************

**XylT rev c Intron site**

gi|77540264|gb|DQ192540.1| ATTCAGTTAGTTGGCGCCG**ATGAATTTCACTGTTCTGAG**TGGATTGA**G**GAGCCGTCACTT

gi|58696563|emb|AJ627182.1| ATTCAGTTAGTTGGCGCCGATGAATTTCACTGTTCTGAGTGGATTGA**G**GAGCCGTCACTT

gi|1027858651|ref|NM_001325611.1| ATTCAGTTAGTTGGCGCCGATGAATTTCACTGTTCTGAGTGGATTGA**G**GAGCCGTCACTT

gi|1027859365|ref|NM_001324669.1| ATTCAGTTAGTTGGCGCCGATGAATTTCACTGTTCTGAGTGGATTGA**G**GAGCCGTCACTT

gi|72388788|gb|DQ119667.1| ATTCAGTTAGTTGGCGCCGATGAATTTCACTGTTCTGAGTGGATTGA**G**GAGCCGTCACTT

************************************************************ ...

**Supplementary Figure S2 (continued).** Alignment of *N. tabacum* β(1,2)-Xylosyltransferase (*XylT*) mRNA, the location of protospacer elements and PCR amplification primers. Protospacer elements are in boxes with arrows showing their direction (< for sense and > for antisense). The protospacer elements are in the format of GN_20_-GG with the first ‘G’ and the Protospacer Adjacent Motif (PAM) sequences NGG highlighted in yellow or grey. Primers for PCR amplification are in bold and underlined. Reverse primers are shown as their reverse-complement counterpart.

**
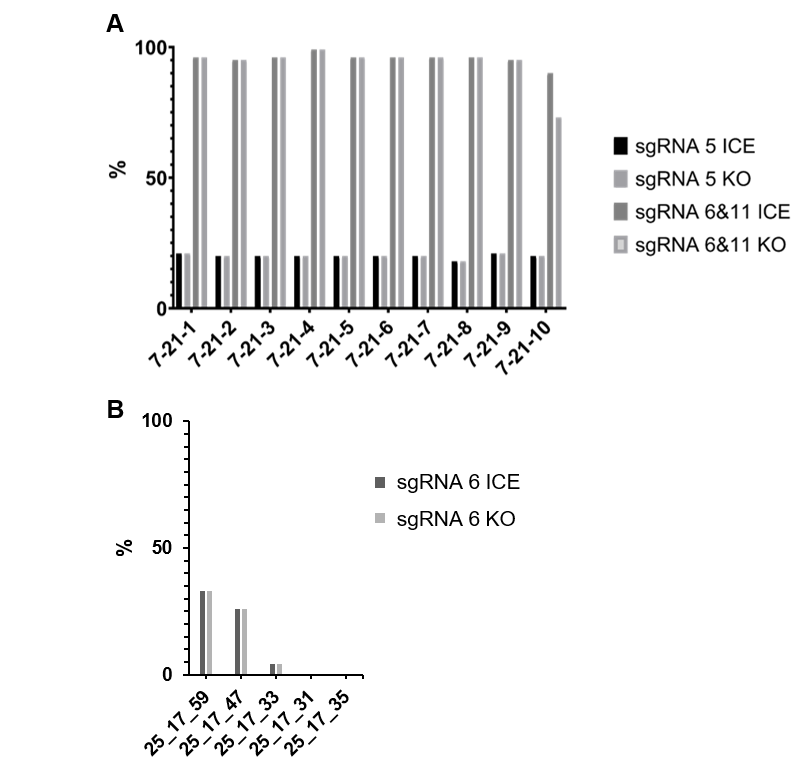
**

**Supplementary Figure S3: Efficiency of *FucT* and *XylT* knockouts using CRISPR/Cas9 constructs pFGC-FucT and pFGC-XylT.** Inference of CRISPR Edits (ICE) and knockout (KO) scores of putative *FucT* knockout plants (**A**; T_2_ generation) and *XylT* knockout plants (**B**; T_3_ generation) were determined using ICE analysis (Synthego) of Sanger sequenced PCR amplicons.

**
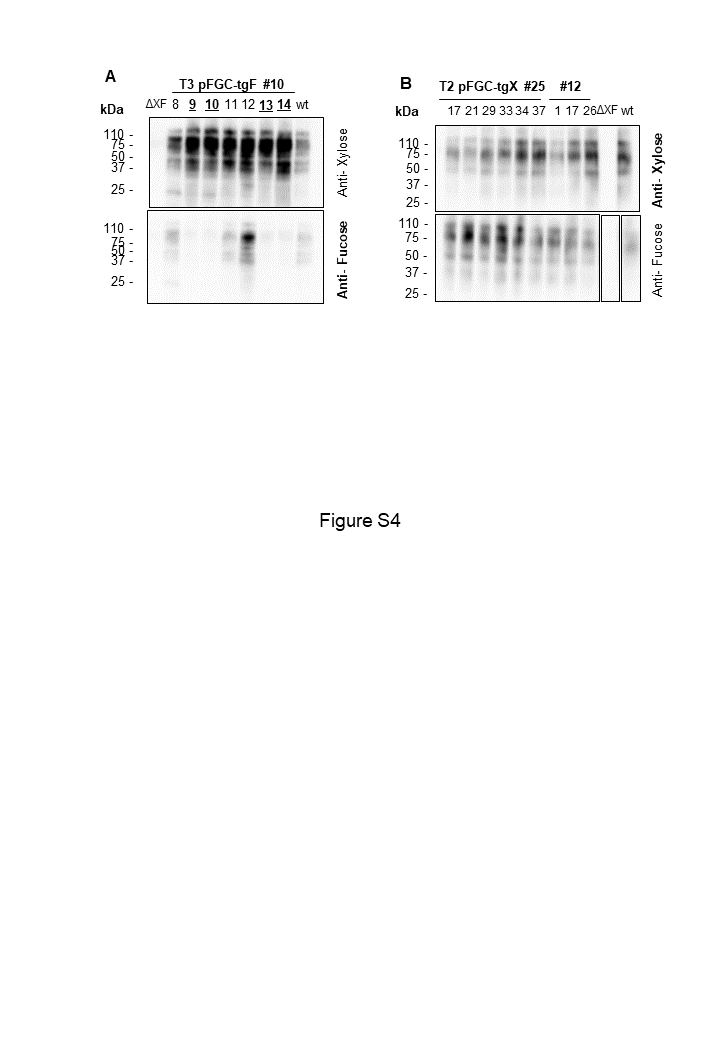
**

**Supplementary Figure S4: Western blot analysis of total soluble protein of putative transgenic *N. tabacum* cv. SR-1 *FucT* (A) or *XylT* (B) knockout lines.** About 10 µg of total soluble protein from wild-type *N. tabacum* cv. SR-1 (wt)*,* putative knockout lines generated by pFGC-FucT (**A**; T3 generation) or pFGC-XylT (**B**; T2 generation), and *N. benthamiana* ΔXF/FT plants (ΔXF; kindly provided by BOKU Vienna), were loaded and incubated with either rabbit anti-α(1,3)-fucose or anti-β(1,2)-xylose antisera followed by HRP-labelled goat-anti-rabbit H+L antisera. Blots for *N-*glycans that were not affected (i.e. anti-xylose blots for *FucT* knockout lines; anti-fucose blots for *XylT* knockout lines; labels not in bold) were used as loading control.
